# Advancing Nitrogen Recovery from Livestock Manure Processing toward a Circular Economy in Agriculture

**DOI:** 10.1021/acs.est.5c18515

**Published:** 2026-05-12

**Authors:** McKenzie Burns, Hanyu Tang, Kai Yang, Zhijie Wang, Rui Wang, Fikile Brushett, Song Jin, Rebecca A. Larson, Mohan Qin

**Affiliations:** † Department of Civil and Environmental Engineering, 5228 University of Wisconsin—Madison, Madison, Wisconsin 53706, United States; ‡ Department of Chemistry, 5228 University of Wisconsin—Madison, Madison, Wisconsin 53706, United States; § Department of Chemical Engineering, Massachusetts Institute of Technology, Cambridge, Massachusetts 02139, United States; ∥ Nelson Institute for Environmental Studies, 5228 University of Wisconsin—Madison, Madison, Wisconsin 53706, United States

**Keywords:** resource recovery, sustainable agriculture, membrane processes, electrochemical treatment

## Abstract

Livestock manure is a concentrated waste stream that poses significant threats to environmental health. One of the major concerns is the large concentration of nutrients. For example, nitrogen discharged by livestock in feces and urine ranges from 80 to 131 Tg N yr^–1^ globally. If harnessed entirely, this nitrogen resource could replace a significant portion of the global demand for fertilizer nitrogen applied to crop fields. However, current manure management practices are inefficient and subject to major losses. In this study, we articulate critical challenges in manure nitrogen management and processing, as well as present an overview of recent advancements in technologies aimed at nitrogen reclamation from livestock manure, including membrane-based technologies and electrochemical techniques. The former achieves excellent total ammoniacal nitrogen recoveries of up to 95%, and the latter can be integrated with membranes or used independently to further enhance nitrogen recovery. We analyze the principles of these novel technologies, present a comprehensive understanding of how they work, and provide a critical evaluation of their strengths and weaknesses. This review provides vital insights on nitrogen recovery from livestock manure, paving the way for a more sustainable future for manure management to achieve a circular economy in agriculture.

## Graphical abstract

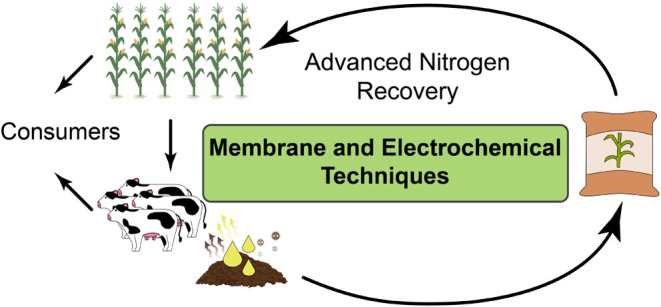

## 1 Environmental Impact of Nitrogen Loss from Livestock Manure Systems

Nitrogen is an essential nutrient in livestock diets and serves as a key fertilizer for crop production. The rate of nitrogen application to crop fields globally has increased significantly over time, rising from an average of 45 kg N ha^–1^ yr^–1^ in the 1960s to 101 kg N ha^–1^ yr^–1^ in the 2010s.^1^ A large portion of the nitrogen applied to cropland comes from livestock manure (from animals both on pasture and in confinement), a mixture of feces, urine, and other system byproducts (e.g., wash waters, waste feed). The global nitrogen excretion from animals is estimated to be between 80 and 131 Tg N annually—greater than the nitrogen used in commercial fertilizers over the same period.^2–6^ More recent assessments indicate values at the higher end, likely due to increasing global animal populations.^6,7^ Although recycling manure nutrients by applying them to cropping systems is sustainable in principle, only 0–60% of manure nitrogen (global mean of 15%) is absorbed by plants, with 40–100% lost to the environment, highlighting the urgent need for intervention (Figure 1).^3^ Global-scale assessments also highlight several hot spots (10% of land receiving 50% of nitrogen inputs) that have high manure nitrogen production, high nitrogen fertilizer application, and high levels of hypoxic zones.^7^ These nitrogen losses are particularly concerning in the forms of nitrous oxide (N_2_O), ammonia (NH_3_), and nitrate (NO_3_^–^), which have significant impacts on the environment.

**Figure 1:**
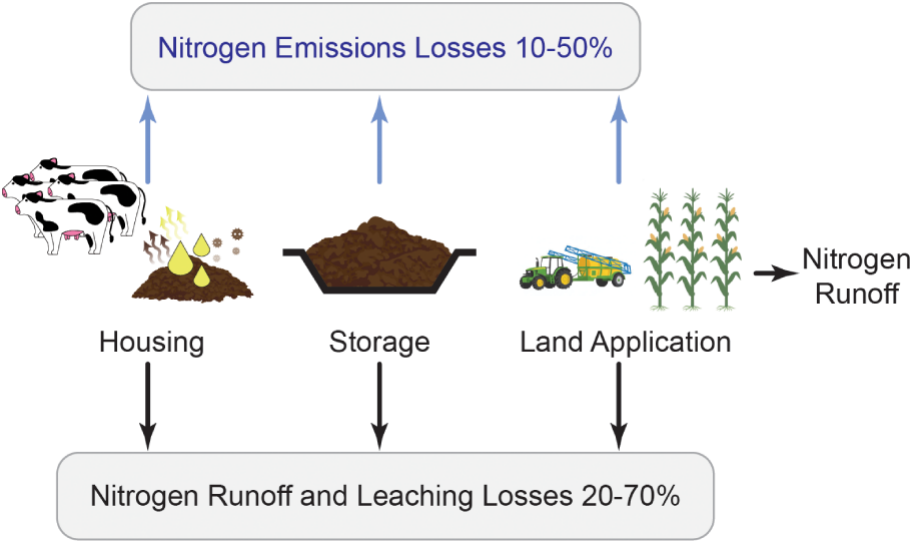
Manure nitrogen loss pathways in livestock agricultural systems.^3,8^

Nitrogen losses as NH_3_ account for approximately 23% of nitrogen losses from manure globally.^9^ Consequently, livestock manure management is the primary source of anthropogenic NH_3_ emissions, representing approximately 50% of NH_3_ emissions in the United States.^10^ Once emitted to the atmosphere, NH_3_ can be converted into N_2_O, a greenhouse gas, or form fine particulate matter (PM_2.5_), which is a known air quality pollutant that poses a significant risk to human health. In addition, atmospheric deposition of nitrogen can increase nitrogen levels in terrestrial and aquatic environments. Global deposition was estimated at 94 Tg N per year in 2016, representing a global average increase of 8% from 1984 to 2016, although there are clear regional differences.^11^ Previous studies have reported that inorganic nitrogen deposition is driven by regional emissions^11,12^ and further highlight improvements in policies related to combustion but not from livestock manure and fertilizer in the United States, driving increases in nitrogen deposition in reduced forms. While total nitrogen deposition decreased in the United States from 2002 to 2017 due to reductions in oxidized nitrogen emissions, largely driven by controls on fossil fuel combustion from power plants and vehicles, increases in deposition of reduced nitrogen forms in the upper Midwest, northern Rocky Mountains, and western United States are attributable to increased precipitation and agricultural emissions.^13^ Furthermore, volatilized NH_3_ reduces the nutrient value of manure, often necessitating additional nitrogen fertilizer production and application. Since synthetic nitrogen fertilizers are energy-intensive to produce and primarily rely on nonrenewable resources, their production further adds to anthropogenic greenhouse gas emissions. Commercial nitrogen fertilizer production, primarily through the energy-intensive Haber-Bosch process, accounts for approximately 5% of global climate emissions annually.^14^

Crop and livestock activities contribute to 10–14% of global greenhouse gas emissions^15^ and approximately 10% in the US.^16^ Nitrous oxide emissions from livestock manure account for approximately 30% of anthropogenic N_2_O emissions globally,^17^ with livestock N_2_O emissions increasing by approximately 40% from 1990 to 2020.^16,17^ In the US, N_2_O from manure management increased from 12.4 to 17.4 million metric tons (MMT) of carbon dioxide equivalent (CO_2_-eq) between 1990 and 2021, accounting for 3% of US agricultural emissions in 2021.^16^

Nitrogen losses from manure via runoff, through leaching, or as NH_3_ deposition into surface waters can impair water quality. Following manure land application (or deposition), nitrogen can undergo mineralization and nitrification, converting organic and ammoniacal nitrogen to nitrate (NO_3_^–^).^18,19^ Although NO_3_^–^ is plant-available, its mobility increases risks of leaching beyond the root zone and contaminating groundwater.^20^ Nitrate leaching beyond the root zone represents up to 30% of nitrogen applied to agroecosystems.^21^ Previous assessments have noted significant increases in NO_3_^–^ groundwater concentrations in areas with high nitrogen inputs from manure and conditions that favor transport to groundwater.^22–24^ Nitrate contamination is especially high in U.S. regions with intensive nitrogen application and shallow groundwater sources.^23^ Reducing post facto groundwater nitrate contamination is extremely challenging and costly; thus, it is critical to reduce leaching from agricultural activities, particularly in areas with high livestock density, shallow groundwater, and high transport potential.

Nitrogen losses from manure occur mainly in three areas on livestock farms, in increasing order of magnitude: (1) livestock housing at 10–90% (high end for poultry and cattle feedlots), (2) storage at 4–70% (highest from anaerobic lagoons), and (3) land application at 1–60% (broadcast application resulting in the largest emissions).^25–27^ Notably, NH_3_ losses tend to be a significant portion of manure nitrogen lost and are influenced by factors such as manure dry matter content, nitrogen content, land application method, temperature, surface area exposure, wind, and pH.^28^ Overall, only 10–30% of nitrogen excreted by the animal moves through the system and is taken up by plants following land application.^3^ Strategies for mitigating losses that have greater potential for mitigating nitrogen losses from livestock systems include animal management and housing (e.g., feeding strategies that reduce protein in feed to reduce nitrogen excretion), manure storage, and improving nitrogen use efficiency of manure as a fertilizer (e.g., injection of manure during land application).^3,29^ However, challenges remain in the integration of manure nitrogen mitigation strategies.

## 2 Critical Challenges in Sustainably Managing Manure Nitrogen

Managing manure nitrogen sustainably is inherently complex due to the diverse composition of manure, the wide range of livestock systems, the need to integrate technological and farm system knowledge, and the farm-scale economic constraints that influence manure system advancements. As manure contains multiple constituents beyond nitrogen, focusing solely on nitrogen management could inadvertently worsen other sustainability metrics. Additionally, manure composition, including the nitrogen content, varies significantly across livestock systems and even within a single farm.^30,31^ This variability necessitates flexible manure nitrogen management strategies that can adapt to shifting nitrogen levels and ratios relative to other components, presenting significant challenges when the manure composition remains unaltered.

Beyond manure variability, manure handling and management practices differ widely among farm systems.^27^ As a result, strategies that improve nitrogen retention and utilization in one system may not yield the same benefits in another.^27,29^ Addressing these differences requires large-scale assessments across multiple farm configurations, allowing individual farms to evaluate their options effectively. While models can facilitate this process, most farm system models available to producers offer limited manure management options.^32^ Furthermore, data on the economic implications of integrating manure nitrogen management systems remains scarce. Since farm processes within one farm operation are highly interconnected, isolating precise financial impacts is difficult—a challenge further exacerbated by the gap between technological expertise and an understanding of farm systems.

Even when practices for enhancing manure nitrogen management are identified, economic feasibility remains a major hurdle.^33^ At the farm scale, manure nitrogen management faces high costs, operational complexities, and underdeveloped markets for recovered manure-based products.^34^ Addressing these costs requires maximizing efficiency and creating market demand for manure-based products to offset expenses and compete with synthetic fertilizers. However, economic comparisons based solely on market prices do not account for environmental externalities associated with nitrogen losses and emissions from synthetic fertilizer production.^35,36^ Watershed-level approaches attempt to integrate local environmental goals but face similar barriers, as field- and farm-scale practices remain the primary mechanisms for nitrogen management.^37^ These barriers include financial constraints, coordination challenges, and the need for advanced methods to quantify and mitigate nitrogen losses.^33^ Effective implementation requires collaboration among livestock farmers, industry stakeholders, citizens, and government agencies, as well as the development of marketing and distribution strategies to enhance system-wide efficiency. Taken together, these challenges indicate that improving manure nitrogen management requires more than optimization of existing on-farm practices; it necessitates reconsidering how manure is processed within the system itself. This shift from addressing management constraints to examining manure processing creates a natural entry point to explore strategies designed to retain nitrogen, convert it to benign forms, or recover it for reuse.

Manure processing strategies for nitrogen management generally fall into three main approaches: retaining nitrogen, promoting nitrogen loss as nitrogen gas, and recovering nitrogen in alternative forms.^38,39^ Retention strategies focus on minimizing nitrogen losses during storage and application by using covers or amendments such as acidification or biochar. While these approaches can significantly reduce emissions during storage, they do not fully prevent nitrogen losses during land application, requiring additional strategies to mitigate field losses. Another approach involves promoting nitrogen loss as nitrogen gas (N_2_) using nitrification–denitrification systems, reducing the nitrogen available to be lost as NH_3_.^40,41^ However, this process can be costly without a mechanism to recover value, and it decreases the nitrogen content of manure, increasing the reliance on synthetic fertilizers. The third approach, nitrogen recovery, seeks to extract and convert manure nitrogen into a reusable form while preserving its nutrient value. While this strategy helps minimize nitrogen losses and improve nutrient management, it requires careful oversight to maintain nitrogen-use efficiency, particularly for the recovered nitrogen. Managing nitrogen losses in manure systems is inherently complex, yet recovering nitrogen for reuse presents an opportunity to mitigate losses from both storage and land application. By extracting nitrogen for fertilizers and other products, these strategies have the potential to reduce the dependence on energy-intensive synthetic nitrogen production.

In many cases, the viability of nitrogen recovery from manure depends on selling recovered products or reducing operating costs to justify the investment. This is particularly challenging when synthetic nitrogen fertilizer is available at low prices, ranging from approximately $0.50 to $2.50 USD per kilogram of nitrogen in the U.S. between 2010 and 2024.^42^ For nitrogen recovery to be financially viable, the value of recovered nitrogen products must at least match the recovery costs. However, current nitrogen recovery technologies vary in cost, with reported costs being highest for struvite precipitation, followed by ion exchange with ultrafiltration (UF), conventional air stripping, membrane distillation, and reverse osmosis with UF.^34^ These reported recovery costs vary widely by technology and scale, ranging from $3.40 to >$26.20 USD per kg of recovered nitrogen, exceeding fertilizer prices, making widespread adoption unlikely under current economic conditions.^34,43^ Comparable recovery costs for novel technologies discussed later in this review (including membrane and electrochemical technologies) are currently unavailable due to the relative novelty and lack of full-scale demonstration of these concepts.

The feasibility of nitrogen recovery also depends on a farm’s specific needs. Farms requiring additional nitrogen must justify recovery costs through increased nitrogen use efficiency, reducing reliance on synthetic fertilizers, whereas those with excess manure nitrogen (occurring on farms with significant feed imports) require revenue from the sale of recovered nitrogen products. In both cases, current recovery technologies are unlikely to be cost-effective without additional incentives.^43^ Furthermore, many recovery methods coextract multiple nutrients at dilute concentrations, thereby increasing transportation and land application costs compared to conventional fertilizers. Therefore, other cost-saving measures, new product markets, or environmental credits are needed to incentivize nitrogen recovery. Analyses of nitrogen impacts indicate that cost–benefit analyses do not thoroughly integrate environmental, economic, and societal outcomes; thus, avoided damages are often not fully considered.^44^ Studies estimate these avoided costs at $32–$35 USD per kg of nitrogen released, which could significantly alter the financial outlook.^43^ Even a small investment in nitrogen recovery could make some technologies economically viable while simultaneously mitigating nitrogen losses to the environment. However, ongoing monitoring is necessary to ensure these outlays remain cost-effective over time.

## 3 Manure Processing Technologies to Recover Manure Nitrogen

Effective recovery of manure nitrogen requires an understanding of its forms, separation mechanisms, and quantification methods. Various conventional and emerging technologies can be integrated into farm systems; selecting the right system depends on the manure type, recovery driver, and technology specifications.

### 3.1 Forms of Nitrogen in Manure

Livestock manure contains both organic and inorganic nitrogen (Figure 2), where the majority of nitrogen is commonly organic but varies with manure type and system.^18,19,45^ Organic nitrogen in manure consists of a vast array of compounds, including proteins, amino acids, and urea. Organic nitrogen can be converted to inorganic forms, such as ammonium (NH_4_^+^), through physical and biological processes. Ammonium is subject to partitioning into ammonia (NH_3_) according to the equilibrium of the NH_3_–NH_4_^+^ system (*pK*_a_ = 9.25 at 25 °C). In manure with a pH above 9.25, the majority of ammoniacal nitrogen is in the form of NH_3_, which can be separated by volatilizing gaseous NH_3_. When pH < 9.25, NH_4_^+^ remains and, as a charged cation, can be further recovered in the liquid fraction of manure.

**Figure 2:**
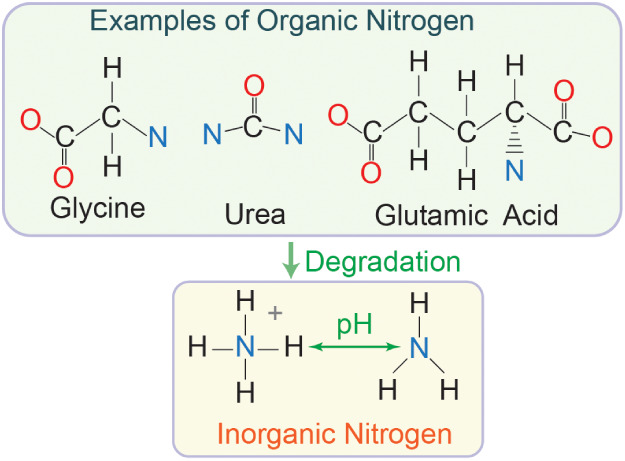
Examples of nitrogen speciation found in manure.

It is important to consider the speciation of nitrogen in manure while selecting and evaluating treatment and recovery processes. As much of the organic nitrogen in manure is consolidated within particulate matter, physical separation systems that effectively remove larger particulates succeed in concentrating organic nitrogen in separated manure solids as a product. Inorganic ammoniacal nitrogen can be recovered in liquid streams from physical separations or targeted through chemical separation, such as NH_3_ stripping and struvite precipitation. Because most nitrogen in the global fertilizer market is in inorganic forms, it is advantageous to recover manure nitrogen in an inorganic form. Such recovery can be achieved through processing to mineralize manure organic nitrogen into inorganic forms before targeted recovery. As microbes are the primary drivers of nitrogen mineralization, biological treatment processes are often effective. Anaerobic digestion, for example, can increase total ammoniacal nitrogen (TAN) concentrations by 6–150%.^46,47^ However, conversion can result in increased losses as NH_3_ unless more effectively managed or recovered.^46,48^ Several novel technologies discussed herein facilitate *in situ* organic nitrogen mineralization via biological and electrochemical processes, enhancing total nitrogen removal.^49,50^ Manure treatment technologies can also be combined in series to remove both organic and inorganic, often ammoniacal, nitrogen. Selecting appropriate system components ensures effective removal and supports the integration of novel nutrient recovery technologies with existing infrastructure, thereby facilitating farm-scale adoption.

### 3.2 Methods for Quantifying Separation Systems

Manure separation systems are among the most widely assessed manure processing systems, with numerous review papers.^38,51–53^ Selecting performance metrics for separation is critical for comparative system evaluations. Two primary metrics used, the separation index (SI) and removal efficiency (RE), can be used to compare performance across variability in manure characteristics.^38,51,54^ The SI, which incorporates mass distributions by estimating the ratio of solids in the effluent fractions to the inputs, is less commonly used because it requires more detailed calculations and the characterization of all separated products. Furthermore, in novel technologies, this calculation is not useful as it incorporates the dry matter of the influent and separated products, as well as the nitrogen concentration, whereas, in the case of more advanced nitrogen removal, the recovered nitrogen product is of high purity. Thus, we examine technologies using the RE (eq 1).

1 $$RE_{x}=1-\frac{{[X]}_{\mathrm{liquid}\;\mathrm{effluent}}}{{[X]}_{\mathrm{influent}}}$$

where *X* is the constituent concentration under evaluation.

### 3.3 Conventional Manure Nitrogen Recovery Technologies

Technologies commercially available for recovering nitrogen from manure include separation systems, NH_3_ stripping, and struvite recovery. Separation systems are among the most widely implemented systems for nutrient recovery from manure, and they have been well documented in previous reviews.^38,47,52,53^ Mechanical-based separation systems (e.g., screens, presses, centrifuges, etc.) are among the most commonly integrated systems in livestock manure processing,^55^ with a recent survey finding that 15% of U.S. dairy farmers surveyed had adopted some mechanical separation systems and that 41% of nonadopters expressed interest or openness to adopt the practice in the next ten years.^56^ While they have improved nitrogen removal efficiencies compared to gravity settling systems, mechanical separation systems are generally considered low-efficiency separation systems for nitrogen.^46,54^ This low efficiency is in part due to the nondiscriminatory nature of mechanical separation when it comes to the recovery of organic and inorganic nitrogen. As different speciations of nitrogen have different affinities for solid and liquid fractions (with organic nitrogen favoring solids and inorganic, often ammoniacal, nitrogen favoring liquids), they are frequently separated into different product streams due to the lack of N transformations within mechanical separation. Among mechanical separation systems, centrifuges have an RE of 28% for nitrogen, drainage systems (e.g., belt press) 27%, and pressurized systems (e.g., screw press) 15% nitrogen.^38^ In all cases, mechanical systems are more effective in removing organic nitrogen, where the RE of ammoniacal nitrogen is significantly less than total nitrogen due to the priority of solid fractions as the main product from these processes. Regardless of the system used, conventional manure separation systems are generally considered to have low separation efficiency.^54^ With low recoveries, it can be difficult to justify the costs of system operation, particularly for nitrogen recovery. In addition, the separated products have a variety of other constituents contained within the product, which can increase the costs of management and transport as well as make comparisons to commercially available fertilizers difficult, hindering market development.

Direct recovery of inorganic ammoniacal nitrogen from manure can be achieved through more specific chemical processes, often focused on the liquid fraction. NH_3_ stripping, which transfers NH_3_ from the liquid phase to gaseous form, has been used to separate dissolved NH_3_ from manure. Although NH_3_ stripping can achieve nitrogen recovery rates of 80–90%, the process requires careful pH control and adequate aeration to operate effectively.^57–59^ This results from the fact that the NH_4_^+^/NH_3_ ratio, which is crucial for the efficiency of the process, is controlled by the pH value of manure (further discussed in Section 4.1). In addition, in raw manure, nitrogen predominantly exists in organic nitrogen forms with a relatively low NH_4_^+^/NH_3_ ratio, which requires prior mineralization to ammoniacal forms for effective recovery with this technology (discussed in Section 3.1). One of the major drawbacks of NH_3_ stripping is its high energy demand, which ranges from 1.5 to 12 kWh_el_ m^–3^ and 62 to 69 kWh_th_ m^–3^, leading to operational costs of approximately $4.9 to $9.4 USD m^–3^.^47^ Struvite precipitation, a process that recovers nitrogen and phosphorus by forming solid MgNH_4_PO_4_·6H_2_O when its ionic concentration exceeds the solubility limit, can also achieve NH_3_ extraction from liquid manure. However, because struvite precipitation is primarily regarded as a phosphorus recovery technology and achieves only limited nitrogen recovery (10–40%), it is not discussed in detail here.^47^ A summary of conventional and novel technologies for nitrogen recovery from livestock manure is included in Table 1. Both nitrogen removal and nitrogen recovery efficiencies are reported where available.

**Table 1:** Summary of Conventional and Novel Technologies for Nitrogen Recovery from Livestock Manure^a^

			**Nitrogen Removal Efficiencies**			**Nitrogen Recovery Efficiencies**		
Classification	Technology	Feedstock	TN	orgN	TAN	TN	TAN	References
Conventional Manure Processing	Centrifuge		12%	50%	8%			^54^ ^d^
	Centrifuge		34%		17%			^53^
	Screw Press		10%	14%	2%			^54^ ^d^
	Screw Press		19%		12%			^53^
	Ammonia stripping					80–90%		^47^
	Struvite precipitation					10–40%		^47^
Novel Membrane Technologies	Reverse Osmosis	concentrated ammonium feed			up to 99%^b^			^60^
	Reverse Osmosis	AnMBR effluent			up to 99.8%^b^			^61^
	Reverse Osmosis	pretreated swine manure			up to 99%^b^			^62^
	Forward Osmosis	cow manure digestate			up to 95%^b^			^63^
	Forward Osmosis	swine manure digested centrate	up to 40%^b^		up to 40%^b^			^64^
	Electrified Ultrafiltration	synthetic swine manure digestate			NH_3_–N: 37% NH_4_^+^–N: 21%			^65^
	Modified Nanofiltration	domestic WW digestate			up to 78%^b^			^66^
	Donnan Dialysis	synthetic WW					90%	^67^
	Donnan Dialysis	concentrated ammonium feed			80%			^68^
	Donnan Dialysis	synthetic urine					66%	^69^
	Isothermal Membrane Distillation	urine					60%	^70^
	Solar Membrane Distillation	synthetic WW			84%^c^			^71^
	pH-adjusted Membrane Distillation	domestic WW			50%			^72,73^
Novel Electrochemical Technologies	Electrodilaysis	synthetic livestock WW			45%^c^			^74^
	Electrodilaysis + Reverse Osmosis	swine manure					67%	^75^
	Electrodilaysis Reversal	swine manure digestate					100%	^76^
	BES—MFC	dairy manure WW	60%	70%				^49^
	BES – 3 chamber MFC	synthetic WW			98%			^77^
	BES – 3 chamber MEC	pig slurry digestate			24%			^78^
	AnOMBR + MEC	synthetic WW					45%	^79^
	Prussian Blue Analogs	domestic WW			85%			^80^
	Selective Redox Material	dairy manure WW			66–68%		68%	^81^

a TN = total nitrogen, orgN = organic nitrogen, TAN = total ammoniacal nitrogen, WW = wastewater, BES = bioelectrochemical system, MFC = microbial fuel cell, MEC = microbial electrolysis cell.

b Reported as rejection.

c Calculated from published values.

d Values are reported as the average.

## 4 Emerging Technologies for Ammonia Recovery

Considering that much of the global fertilizer market is reliant on inorganic ammoniacal nitrogen, the novel technologies discussed here focus on the separation and recovery of total ammoniacal nitrogen (TAN). Some technologies, specifically those that use electrochemical reactions and conversions, can enable additional nitrogen recovery through the *in situ* mineralization of organic nitrogen in manure into inorganic ammoniacal nitrogen. Here, we discuss the factors affecting the separation and recovery of TAN and provide an overview of recent studies that exemplify novel technologies for the recovery of nitrogen from livestock manure. A summary of the novel technologies and their nitrogen removal and recovery efficiencies is reported in Table 1.

### 4.1 Separation Mechanisms of TAN

A variety of separation mechanisms based on pH, solubility, molecule size, volatility, charge, reduction potential, and diffusivity of TAN have been investigated to achieve nitrogen recovery from livestock manures (Figure 3). Specific technologies often rely on multiple separation mechanisms to achieve maximum nitrogen recovery. Here, we discuss the fundamental theory behind each separation mechanism and how these mechanisms drive TAN recovery. Membrane-based technologies primarily select molecules based on either size or charge. In size exclusion, the smaller NH_3_ molecule is permitted through the membrane pores, while larger molecules, including NH_4_^+^ are excluded (Figure 3A-1). Size exclusion is the main separation mechanism in pressure- and thermal-driven membrane processes. In charge exclusion, charged species (such as NH_4_^+^) are transported through membrane pores lined with oppositely charged functional groups, while neutral (such as NH_3_) and similarly charged molecules are excluded (Figure 3A-2). Charge exclusion is the main separation mechanism in concentration-driven membrane processes, as well as those that incorporate electrochemical and membrane technologies to achieve N recovery.

**Figure 3:**
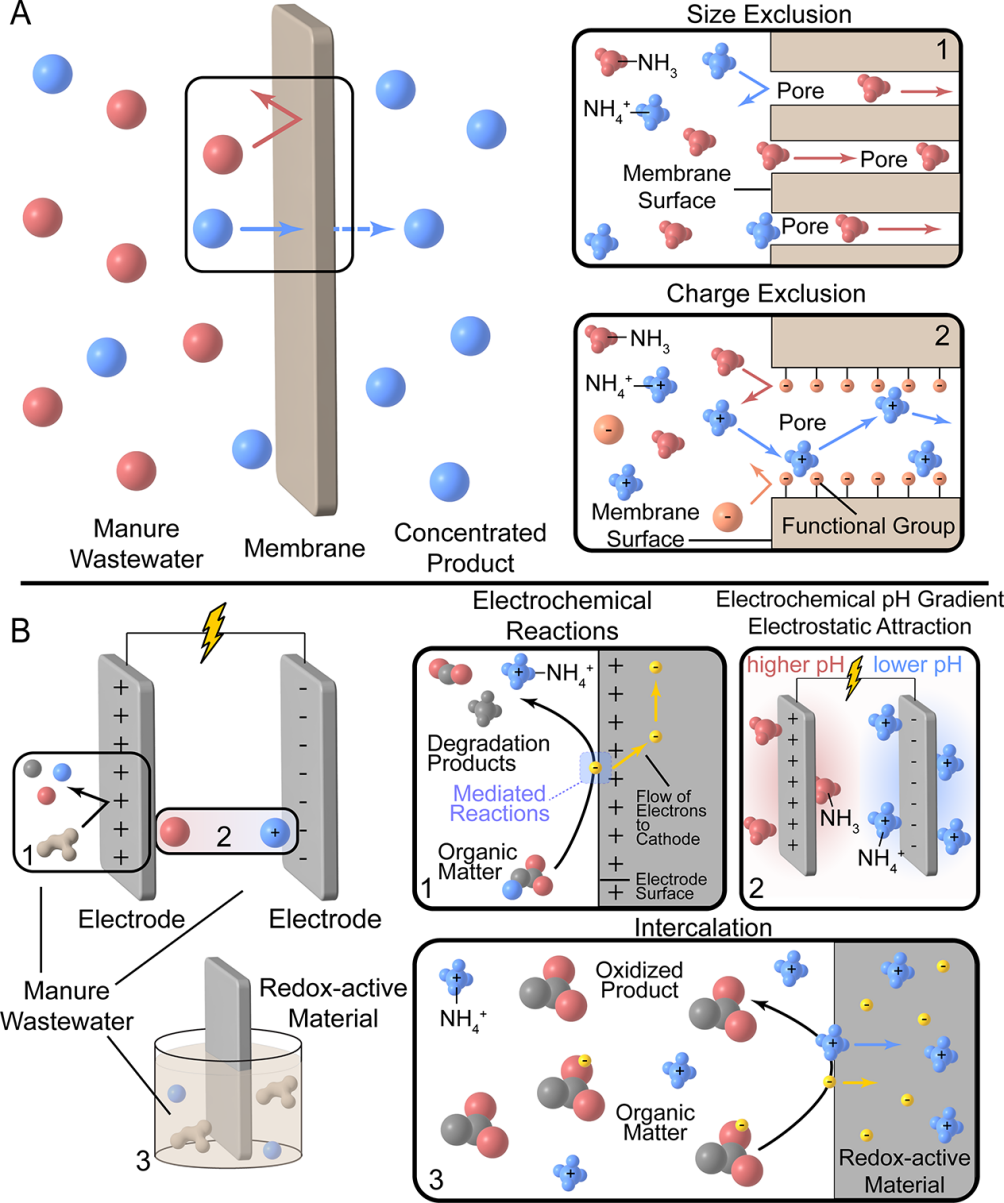
Separation mechanisms and operational principles that underlie novel technologies for TAN recovery from livestock manure. (A) Membrane-based technologies select certain molecules based on either size or charge. (B) Electrochemical technologies rely on the principle of the reduction potential of chemical species to select specific molecules or ions.

Electrochemical technologies rely on the principle of the reduction potential of chemical species to select specific molecules or ions. Electrochemical reactions (Figure 3B-1) transform certain reactant molecules into target degradation products through the accumulation or loss of electrons at an electrode, such as the production of NH_4_^+^ from the oxidation of organic nitrogen compounds. These reactions occur at the electrode surface and can be mediated by chemical or biological agents. Electrochemical pH gradients (Figure 3B-2) are generated due to reactions occurring at the electrode surface and can help to select specific molecules (e.g., NH_3_ or NH_4_^+^) based on the p*K*_a_ of the acid–base system. Additionally, electrostatic attraction drives ions toward oppositely charged electrodes, such as in the electrosorption of the positive NH_4_^+^ on a negatively charged electrode. Intercalation (Figure 3B-3) uses redox-active materials to oxidize organic matter while uptaking both electrons and positive ions, such as NH_4_^+^, into the redox material matrix. The redox material can later be discharged, releasing the recovered NH_4_^+^.

System pH value plays an important role in TAN recovery as it governs the speciation of ammoniacal nitrogen. The charged NH_4_^+^ ions dominate at pH values below the p*K*_a_ of 9.25. The positive charge and larger ionic radius (1.48 Å) make charge and size exclusion effective separation mechanisms for NH_4_^+^ removal (Figure 3A). Furthermore, the charge of NH_4_^+^ permits participation in electrochemical separations such as ion intercalation (Figure 3B-3). Above the pH of 9.25, the neutral NH_3_ molecule dominates, permitting its transport through both hydrophobic and charged membrane pores (Figure 3A) and allowing for separations based on the volatility of gaseous NH_3_ (K_H_ = 62 M atm^–1^ at 25 °C). Solubility is another factor that impacts ammoniacal nitrogen recovery from manure. As both forms of ammoniacal nitrogen are quite soluble in water at standard conditions (520 g L^–1^ for NH_3_ and 10.2 g L^–1^ for NH_4_^+^, both at 20 °C), precipitation-based technologies (such as struvite precipitation described in Section 3.3) are usually ill-fitted for TAN recovery alone. Diffusivity, or the ease with which molecules move through membranes, is relevant to any technology employing membrane separation. Lastly, the electrochemical reduction potential, defined as the tendency of a chemical species to gain or lose electrons, typically at an electrode, plays a role in electrochemical recovery technologies in which nitrogen species undergo redox reactions that govern cell processes (Figure 3B).

### 4.2 Membrane-Based Technologies for Ammonia Recovery

Membrane technologies provide an effective approach to manure treatment by enabling the selective removal and recovery of TAN fractions from manure. The transport of NH_4_^+^/NH_3_ for separation and recovery can be driven by a concentration, pressure, thermal, or electrochemical gradient across the membrane. Membrane processes, including reverse osmosis (RO), Donnan dialysis, and membrane distillation, have been effectively implemented for NH_3_ recovery from livestock manures, although much of the work remains at the laboratory and pilot scale, with limited data available outlining the potential economic impact of these recovery technologies at scale. Electrochemical potential-driven membrane processes will be discussed in Section 4.3.

#### 4.2.1 Pressure-Driven Membrane Processes

In pressure-driven membrane processes, size exclusion is one of the main separation mechanisms. Size exclusion relies on the separation of molecules of certain sizes (those larger than the pore size of the membrane) being excluded from the permeate. Depending on the target molecule’s size, either the permeate, the retentate, or both can be considered the product streams from pressure-driven membrane processes. Ultrafiltration (UF), nanofiltration (NF), and reverse osmosis (RO) are the most frequently employed pressure-driven membrane processes for ammonia recovery. UF membranes have a molecular weight cutoff (MWCO) of 1–500 kDa, and NF membranes have an MWCO of 0.15–0.3 kDa.^82^ UF is usually applied as a pretreatment process, removing suspended solids and large particulates to produce a relatively clean stream for downstream NF or RO for NH_3_ recovery.^62,83–85^ NF rejects most of the dissolved organic matter as well as multivalent ions while allowing monovalent ions and neutral small molecules (e.g., NH_4_^+^ and NH_3_) to pass. For example, a polyelectrolyte-modified NF membrane exhibited good NH_3_–N/organic carbon selectivity.^66^

Reverse osmosis can be used to further concentrate the ammonia-rich NF permeate, enabling the simultaneous recovery of clean water and a concentrated nitrogen product^86^ (Figure 4A). The TAN retention in the concentrate stream was reported to be as high as 99%.^62,83,84^ The feed pH is often a major factor in the effectiveness of both NF and RO membranes for the recovery of TAN, as pH variation changes the membrane charge density and the NH_4_^+^/NH_3_ speciation. In commercial NF and RO membranes, the thin-film composite polyamide active layer plays a critical role; the carboxyl functional groups within the layer deprotonate under neutral and alkaline pH conditions, imparting a negative charge to the membrane.^87,88^ This enhances the rejection rate for all charged ions (e.g., NH_4_^+^). Interestingly, when the pH exceeds 8, the proportion of uncharged NH_3_ in the TAN increases rapidly, while the proportion of NH_4_^+^ decreases. Because NH_3_ is neutral, it permeates the polyamide active layer more readily, reducing TAN rejection rates.^89^ As the feed solution becomes more acidic, the decline in membrane charge density reduces ion rejection. Although TAN predominantly exists as NH_4_^+^ under acidic conditions, excessively low pH also reduces TAN rejection.^90^ Therefore, adjusting the pH can enable nitrogen to pass through the NF membrane more effectively, while the subsequent RO membrane retains it almost entirely.^62^ However, based on the analysis above, RO can achieve optimal TAN retention (99%) only within a narrow pH range close to neutral.^61,91^ Hydraulic pressure, another common operating parameter in pressure-driven membrane processes, primarily determines permeate flux but has little effect on TAN rejection in RO.^60^ For varying feed TAN concentrations (from 55 to 9545 mg N L^–1^) in RO, the permeability coefficient (4.2 × 10^–4^ m h^–1^) and the rejection rate (∼99%) were also found to be relatively stable with little variation.^60^

**Figure 4:**
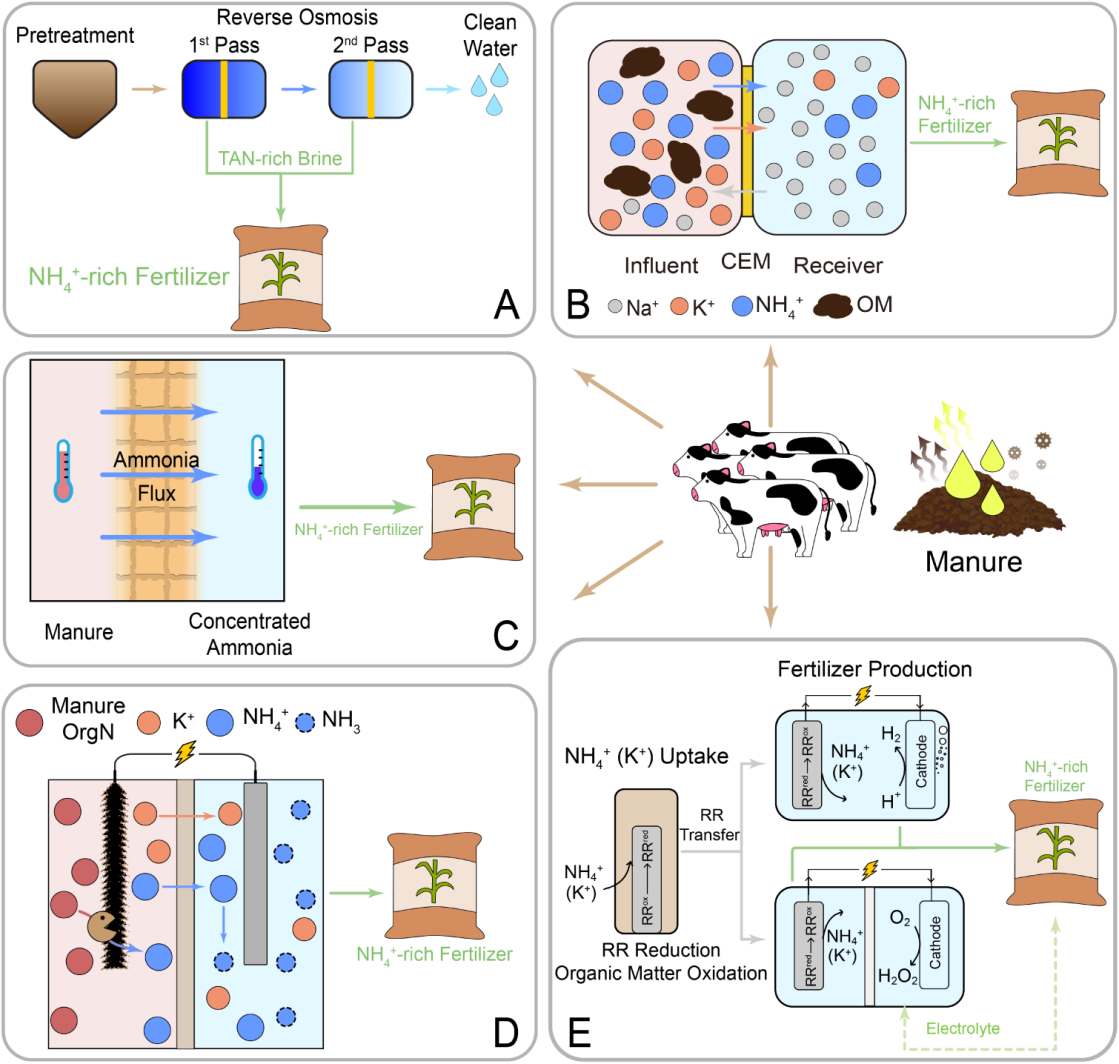
Schematic illustrations of representative technologies for nitrogen recovery from livestock manure, including reverse osmosis (A), Donnan dialysis (B), membrane distillation (C), bioelectrochemical systems (D), and ammonium intercalation (E).

#### 4.2.2 Concentration-Driven Membrane Processes

In concentration-driven processes, the transport of a constituent across a membrane is primarily controlled by diffusion due to a concentration gradient.^92,93^ Concentration-driven processes can employ a variety of membranes depending on the target chemical species or component to be recovered. Two concentration-driven membrane processes frequently implemented for TAN recovery from livestock manure are Donnan dialysis and forward osmosis.

Donnan dialysis (DD) is a concentration-driven membrane process that uses ion exchange membranes (IEMs) as separators between chambers, allowing the permeation of ionic species.^94^ The functional groups in IEMs selectively allow counterions to pass through while rejecting co-ions and neutral molecules.^95,96^ NH_4_^+^ has been effectively recovered from wastewater using the DD process, with recovery efficiency influenced by the cation species in the receiving solution (Figure 4B). The highest recovery rate reported in the DD process is 90% for NH_4_^+^ .^67^ Ammonium recovery rates were studied using flat-sheet and hollow-fiber modules, with 20 mM NH_4_^+^ as the feed solution and 200 mM Na^+^ as the receiver solution, and fluxes of approximately 1 mol m^–2^ h^–1^ were achieved across varying flow rates for both module types.^68^ Furthermore, nutrient recovery from synthetic urine using a DD process with a tubular IEM was shown to enhance recovery efficiency compared with conventional DD setups, achieving 65.6% ammonium recovery from 30 L of synthetic urine, along with other solid products.^69^ The DD process is often combined with other technologies to enhance recovery efficiency by creating a continuous concentration gradient between the chambers.^97^ Building on the concept of electrochemical gradients between chambers, other technologies enhanced by electric fields have been developed and applied to recovery processes.

Forward osmosis (FO) is another concentration-driven membrane process that has been used for TAN recovery from livestock manures. Although FO membranes are similar in structure and composition to RO membranes, the FO process differs from RO in that it relies on the osmotic gradient between the feed and draw solutions, as opposed to a pressure gradient in RO processes. FO processes yielded variable results when used for TAN recovery from manure feed. For example, one study reports an excellent TAN rejection of over 95.5% in aquaporin-based FO membranes with a variety of draw solutions and feed solution pH levels.^63^ However, another recent investigation concludes that aquaporin-based and traditional FO membranes are ineffective at TAN retention, with only approximately 40% rejection of nitrogen species.^64^ Such variation in results from two similar studies could come from different operational feed pH values—the former study operates at a higher feed pH, leading to more NH_3_ species in solution, which are better rejected by the FO membrane.^64^ However, the variation in published results for applications of FO for TAN recovery warrants further independent studies.

#### 4.2.3 Thermal-Driven Membrane Processes

Thermal-driven membrane processes typically rely on thermal energy to induce the phase change of volatile molecules at the surface of the liquid and the gas-permeable membrane, enabling the transmembrane transport of target molecules and retaining nonvolatile ions and molecules. Membrane distillation (MD) is a thermal-driven membrane separation process that has been extensively studied for desalination and shows promise for NH_3_ separation.^98,99^ The gas-permeable membrane in the MD process blocks nonvolatile wastewater streams and allows gas molecules to transport across the membrane, hence enabling NH_3_ gas molecules to be recovered on the other side of the membrane.^100^ The MD process leverages the volatility of NH_3_ and the high pH value of manure toward NH_3_ recovery (Figure 4C). Polytetrafluoroethylene (PTFE), polypropylene (PP), and polyvinylidene fluoride (PVDF) are commonly used membranes for NH_3_ recovery because of their hydrophobicity and their thermal and chemical resistance.^101^ Various types of membrane distillation using gas permeable membranes have been applied for NH_3_ recovery, including vacuum membrane distillation (VMD), sweeping gas membrane distillation (SGMD), direct contact membrane distillation (DCMD), and hollow fiber membrane contactor (HFMC). To enhance the DCMD process performance and reduce heat energy costs, a novel configuration—isothermal membrane distillation with an acidic collector—has been used to improve NH_3_ recovery from urine.^70^ Compared to a conventional configuration, it saves 95.2% of heat energy and recovers approximately 60% of TAN.^70^ Solar irradiation has been introduced to heat the MD solution by incorporating a photothermal effect at the gas permeable membrane surface, which is equivalent to heating the solution by over 20 °C.^71^ A preliminary economic profit analysis of using membrane distillation for NH_3_ recovery from real dairy cattle urine was conducted, and the simulated optimized NH_3_ recovery efficiency reached 98.72%.^72^ In addition, a computational fluid dynamics (CFD) model has been developed to predict liquid flow and mass transfer to derive a more precise permeation rate and recovery ratio of NH_3_ by MD.^102^ These lab-scale experiments provide insights into the feasibility of using MD in livestock farms to recover NH_3_ and for scaling up MD with further commercialization.

### 4.3 Electrochemical Technologies for Ammonia Recovery

Electrochemical approaches have also been applied to the extraction of NH_3_ from livestock manure. Electrochemical approaches to NH_3_ recovery can be roughly classified into three categories: those based on electrochemical reactions, those incorporating electroactive microorganisms to help drive cell processes, and those depending on the intercalation of the target ionic species within the electrode material (Figure 3B). Oftentimes, electrochemical technologies also incorporate membrane processes to enhance the selectivity of the treatment process. Similar to the status of membrane-based technologies for nitrogen recovery from manure, little work has been focused on the potential economic impact of electrochemical technologies for nitrogen recovery from manure, except for a preliminary techno-economic analysis of electrochemical ammonia recovery based on lab-scale experiments.^81^ Again, this is largely due to the lack of operational full-scale systems (as discussed in Section 2).

#### 4.3.1 Electrochemical Reaction-Driven Processes

Electrochemical reactions involve a chemical species gaining or losing negative charge to/from an electrode in an electrochemical cell. While spontaneous (e.g., Galvanic) reactions are possible, more frequently, an applied potential difference between electrodes is used to facilitate electrochemical reactions, driving the movement of TAN within the electrochemical cell. H^+^ ions are generated through oxidation reactions at the anode, decreasing the pH value, while OH^–^ ions are generated through reduction reactions at the cathode, raising the pH value. This polarization in localized pH can lead to the transformation of TAN species within the cell based on the equilibrium of the NH_3_/NH_4_^+^ system (*pK*_a_ = 9.25), which can influence TAN recovery based on cell configuration (Figure 3B-2). Furthermore, when an IEM is implemented, positively charged NH_4_^+^ is transported across the cation exchange membrane to the cathode chamber (where the pH value is elevated) to maintain the charge balance, and NH_4_^+^ ions are converted to uncharged NH_3_. Such applications of electrochemical stripping have been applied to nitrogen recovery from urine and domestic wastewater with high nitrogen recovery efficiencies (near 93%) and show similar promise in applications for nitrogen recovery from livestock manure.^103^

When an electrolysis cell with a high current density (125 mA cm^–2^) was implemented for NH_4_^+^ recovery in synthetic and real livestock wastewaters, a maximum TAN migration efficiency across a cation exchange membrane (CEM) of 44.5% was achieved.^74^ Furthermore, electrodialysis (ED) can be combined with RO processes for the production of a concentrated nitrogen fertilizer, with one study reporting NH_3_ recovery from swine manure of nearly 67% in the RO concentrate.^75^ Further investigation of this technology for NH_3_ recovery sheds light on problematic membrane fouling. Anion exchange membranes were found to foul irreversibly at higher rates than cation exchange membranes in ED cells used for ammonia recovery from swine manure, with calcium hydroxide scaling accumulating on the membrane surface.^84^ In contrast, organic molecules were more likely to foul the cation exchange membrane and could easily be removed with physical cleaning. This is promising, as the cation exchange membrane separates and recovers NH_4_^+^. Similar fouling trends are observed in applications of electrodialysis reversal (EDR), although bench-scale EDR for recovery of nutrients from swine manure digestate can achieve nearly 100% recovery of NH_4_^+^ into the product solution.^76^ Furthermore, in anion exchange membranes mass, conductivity, and ion exchange capacity were found to decrease (likely due to fouling), while no changes in these parameters were observed in cation exchange membranes. However, after several cycles of EDR, growth of irreversible fouling became insignificant, suggesting the long-term feasibility of this technology for nutrient recovery from animal manures.^76^

#### 4.3.2 Bioelectrochemically-Driven Processes

Bioelectrochemical systems (BES) utilize specialized microorganisms to produce electricity and drive electrochemical processes within the cell.^104–109^ These microorganisms, termed exoelectrogens, oxidize organic matter to produce extracellular electrons, which are then transferred to and between electrodes to create current in the cell. BES can take many configurations, of which microbial fuel cells (MFCs) and microbial electrolysis cells (MECs) are most commonly used for ammonium recovery (Figure 4D). In these configurations, cation exchange membranes are used to separate electrode compartments, permitting transport of NH_4_^+^ between chambers for selective recovery.^110–112^ As these BES are electrochemical cells at their core, the same pH changes due to reactions at the electrodes work together to enhance TAN recovery, as described in Section 4.3.1. A significant advantage of incorporating biological processes with electrochemical techniques for nitrogen recovery from livestock manure is the ability to harness organic nitrogen mineralization, carried out inherently by many taxa of microorganisms, to recover organic nitrogen in a mineralized, marketable format. One study used an MFC to recover NH_3_ from synthetic livestock manure, reporting 40–60% removal of total nitrogen when the feed contained organic nitrogen as the main nitrogen source.^113^ Further studies by the same authors applied MFC technology to various compositions of real dairy manure, achieving TN removals of approximately 60% and highlighting organic nitrogen removals of approximately 70%.^49^ In a modified MFC with a third chamber for nutrient concentration and recovery, NH_4_^+^ removal from synthetic wastewater reached 98%.^77^

Several studies have used MECs for ammonia recovery, as the supplemental current applied to the system has been shown to increase NH_4_^+^ ion transport due to electromigration. For example, a three-chambered MEC for recovery of ammonia and phosphate (as precipitated struvite) from digested pig manure slurry achieved a maximum average NH_4_^+^ removal efficiency of 20 ± 4% and reported that NH_4_^+^ transport to the center chamber represented a maximum of 43% of positive charges recovered in the concentrated nutrient solution.^78^ Other studies investigate the incorporation of hydrophobic membranes with MECs. When hydrophobic membrane modules are used for catholyte recirculation, NH_4_^+^ transfer across the CEM increases from 0.26 g N m^–2^ h^–1^ to 0.36 g N m^–2^ h^–1^.^114^ Finally, BES can be integrated with other microbial treatment technologies, such as anaerobic osmotic membrane bioreactors, to enhance nutrient removal and recovery. Such a system recovered up to 45% of TAN in concentrated nutrient solution with minimal fouling.^79^

#### 4.3.3 Redox-Active Materials

Redox-active materials are increasingly attractive for the electrochemical recovery of NH_4_^+^ ions due to their ion selectivity, fast redox kinetics, sustainability, tunability, and compatibility with a variety of electrolyte compositions. To improve the energy efficiency and sustainability of electrochemical ammonia recovery, selective ammonia recovery using redox-active materials has been developed to recover NH_4_^+^ over other cations in various types of wastewater.^80,81,115,116^ Compared to membrane-based processes, selective ammonia recovery provides opportunities to recover nutrients without expensive ion-exchange membranes and offers further opportunities for integration with other chemical production for sustainable agriculture. For example, Prussian blue analogs (PBAs), stable battery materials with open-framework structures, have been studied as the model material for the selective intercalation of NH_4_^+^ and other cations in aqueous solutions.^117–119^ The ion selectivity of the intercalation process arises from the well-defined porous structures of PBAs and the varying sizes of cations. Due to its smaller Stokes radius and desolvation energy, the (de)­intercalation of NH_4_^+^ should be intrinsically faster than that of other metal cations in wastewater. A cation exchange membrane modified with PBA copper hexacyanoferrate (CuHCF) showed a selectivity of over 5 for NH_4_^+^ over Na^+^ in synthetic wastewater with a high initial Na^+^/NH_4_^+^ concentration ratio of 4.^118^ Similarly, a symmetric device with two CuHCF electrodes separated by an anion exchange membrane successfully recovered NH_4_^+^ over Na^+^ with an NH_4_^+^/Na^+^ selectivity above 4 in domestic wastewater and achieved NH_4_^+^ removal of ∼85%.^80^ Furthermore, a stepwise electrochemical process based on the symmetric device could concentrate NH_4_^+^ to 32 mM with an energy cost of 2.0 kWh per kg-N, which was much less than that of capacitive deionization or electrodialysis processes.^120^ The CuHCF electrode also showed good stability for NH_4_^+^ recovery in actual industrial wastewater containing methanol, indicating the possibility of using low-cost redox-active materials for selective nutrient recovery from wastewater.^115^

Using NH_4_^+^-selective redox-active materials to recover nutrients and pairing nutrient production with membrane-free electrochemical synthesis can provide a cost-effective way to further improve the sustainability of nutrient management. Recently, an integrated ammonia recovery and electrochemical synthesis system that involved spontaneous NH_4_^+^ uptake from manure, fertilizer production, electrochemical production of H_2_ or H_2_O_2_, and wastewater treatment was developed (Figure 4E).^81,116^ During the spontaneous process, the reduction of potassium nickel hexacyanoferrate (KNiHCF, another PBA) achieved NH_4_^+^ and K^+^ uptake with a selectivity of ∼100%, driven by the spontaneous oxidation of organic matter in manure. Then, the oxidation of KNiHCF released recovered cations to produce NH_4_^+^ or K^+^ rich fertilizers, paired with the cathodic production of green hydrogen as fuel or H_2_O_2_ as a disinfectant. Nearly 70% of NH_4_^+^ was removed from the manure during the spontaneous uptake process, and all intercalated NH_4_^+^ was eventually recovered after release. The preliminary analysis showed that the electrochemical approach could reduce NH_3_ emissions by up to 70% compared to conversion scenarios in a modeled 1000-cow dairy farm, generating a profit of approximately $200k annually.^81^ An unusual feature of this integrated ammonium recovery process is that the oxidation of the organic matter present in manure, such as urea, sugars, amino acids, and other metabolites, by the redox-active materials drives the selective intercalation of NH_4_^+^, which was removed from the manure during the spontaneous uptake process.All intercalated NH_4_^+^ was then eventually recovered after release of the ions from the redox-active materials (Figure 3B-3).^116^ This process takes advantage of the available organic waste in manure and is distinctively different from other membrane-based or electrochemical recovery processes discussed in this section (see Figure 3B).

## 5 Environmental Implications

This review illustrates the necessity behind effective and efficient nitrogen recovery from livestock manure; however, current manure management practices are inefficient at nitrogen separation and recovery despite high capital and operational costs. Advanced technologies for manure treatment and nitrogen recovery, such as membrane-based and electrochemical techniques, are emerging as promising approaches because of their higher recovery efficiency and long-term sustainability. However, significant limitations of these nascent technologies persist. This review summarizes the recent advances, explains the underlying physicochemical mechanisms, and compares the pros and cons of these emerging advanced technologies. Based on the information provided in this review, a subjective evaluation of 6 of the presented technologies in terms of four merit categories (operational simplicity, ease of scalability, on-farm compatibility, and technology readiness level, or TRL) is summarized in Figure 5. While no single technology can fully address the challenges of nitrogen recovery from manure, there are ample opportunities for further development. For example, selective membranes can be expensive to fabricate, requiring both specialized materials and advanced chemistry expertise. Additionally, all membranes suffer from instances of irreversible fouling, requiring frequent cleaning and eventual costly replacement. For the new redox-active intercalation process, understanding the electron transfer pathway and spontaneous NH_4_^+^ uptake mechanism is critical to designing more effective and robust redox-active materials for nitrogen recovery. Major challenges of electrochemical technologies for manure treatment arise on the financial and scale-up fronts.

**Figure 5:**
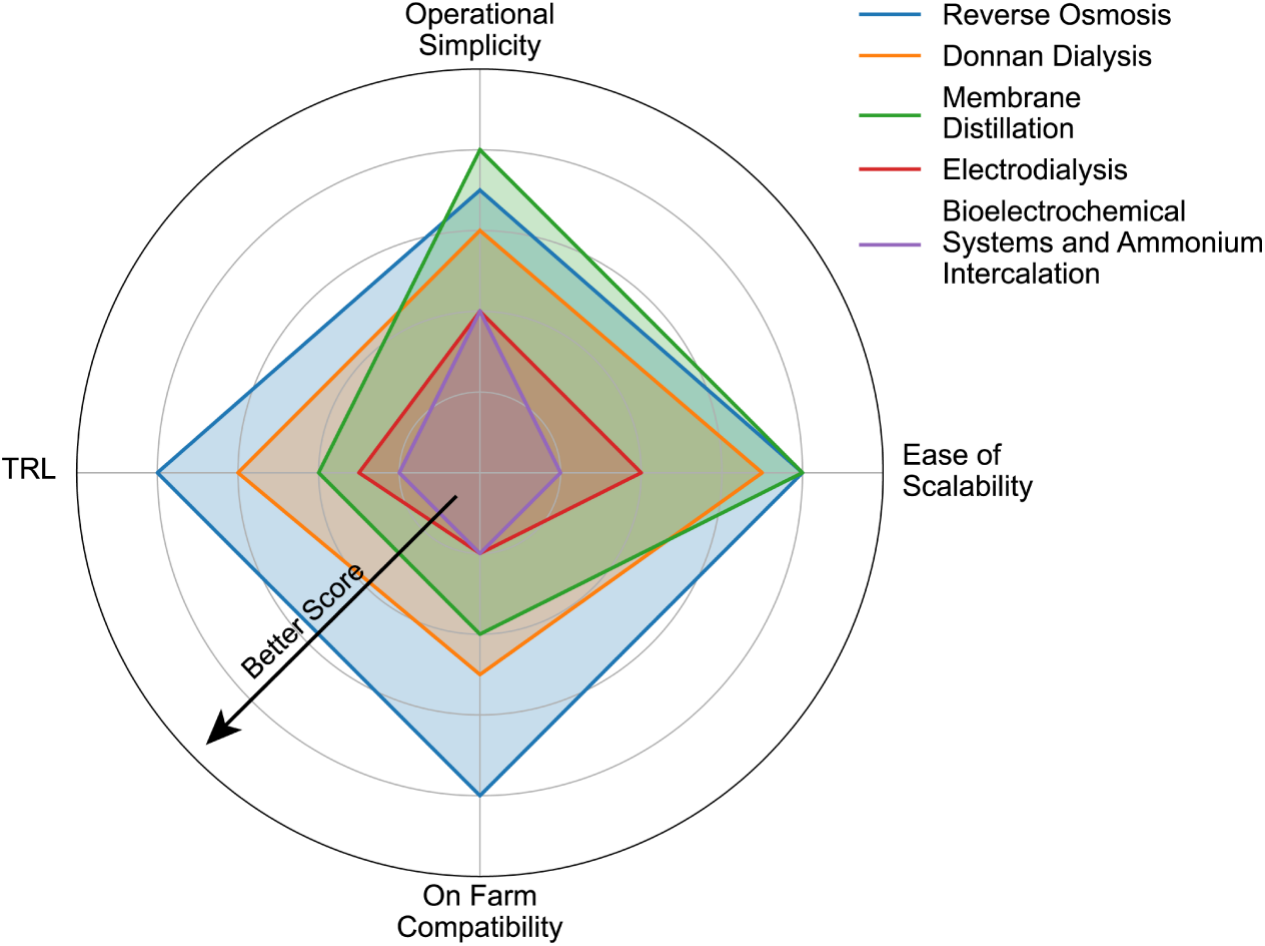
Subjective evaluation of six advanced technologies for nitrogen recovery from manure in terms of four merit categories (Operational Simplicity, Ease of Scalability, On-Farm Compatibility, and Technology Readiness Level or TRL) based on factors discussed in this review. The farther from the center/a larger shaded area indicates a better score. Note that two of six technologies have the same score, so are represented by a single color.

Since manure management is a relatively new application of electrochemical technology, much of the current work is conducted at the laboratory scale, challenging the extrapolation of process efficiencies and cost estimates to the industrial scale. Furthermore, specialized electrode materials face the same hurdles as selective membranes with regard to cost and the expertise required for fabrication.^121^ Because electrochemical approaches are driven by electrical energy, they could also incur high energy costs during operation, which may make them cost-prohibitive for industrial adoption. Despite these barriers, we believe that advanced nitrogen recovery through membrane-based and electrochemical techniques is a worthwhile investment for the future of livestock manure management, especially as distributed renewable electricity continues to become more available and affordable. Technologies focused on circularity are increasingly attractive for their ability to repurpose waste streams as valuable resources, minimizing the ecological footprint of human civilization, reducing the strain placed on biocapacity, while avoiding the substantial health and climate costs of technologies associated with strain on environmental resources. Advancements in technology, especially in novel materials and energy efficiency for renewable energy sources, have the potential to allow us to overcome some of the challenges of membrane and electrochemical technologies in the near future. Therefore, investing in further development of these advanced technologies for nitrogen recovery from manure promises to be a worthwhile venture. As the global population continues to climb, humanity’s impact on the environment remains a grave concern with respect to both resource use and waste output. Implementing advanced treatment for nutrient recovery from livestock manure aims to create a circular economy in agriculture, reducing the environmental and economic impacts of one of society’s largest and most vital industries.
